# Characterization of Norovirus and Other Human Enteric Viruses in Sewage and Stool Samples Through Next-Generation Sequencing

**DOI:** 10.1007/s12560-019-09402-3

**Published:** 2019-08-24

**Authors:** Sofia Strubbia, My V. T. Phan, Julien Schaeffer, Marion Koopmans, Matthew Cotten, Françoise S. Le Guyader

**Affiliations:** 1 Ifremer, Laboratoire de Microbiologie, LSEM-SG2M, BP 21105, 44311 Nantes Cedex 3, France; 2 Department of Viroscience, Erasmus MC, University Medical Center Rotterdam, Rotterdam, The Netherlands; 3 London School of Hygiene and Tropical Medicine, London, UK; 4 Uganda Virus Research Institute, Entebbe, Uganda; 5 MRC-Centre for Virus Research, Glasgow, UK

**Keywords:** Human enteric viruses, Norovirus, Sewage, Metagenomic, Virome

## Abstract

This study aimed to optimize a method to identify human enteric viruses in sewage and stool samples using random primed next-generation sequencing. We tested three methods, two employed virus enrichment based on the binding properties of the viral capsid using pig-mucin capture or by selecting viral RNA prior to library preparation through a capture using the SureSelect target enrichment. The third method was based on a non-specific biophysical precipitation with polyethylene glycol. Full genomes of a number of common human enteric viruses including norovirus, rotavirus, husavirus, enterovirus and astrovirus were obtained. In stool samples full norovirus genome were detected as well as partial enterovirus genome. A variety of norovirus sequences was detected in sewage samples, with genogroup II being more prevalent. Interestingly, the pig-mucin capture enhanced not only the recovery of norovirus and rotavirus but also recovery of astrovirus, sapovirus and husavirus. Documenting sewage virome using these methods provides information for molecular epidemiology and may be useful in developing strategies to prevent further spread of viruses.

## Introduction

Increasing human population leads to increased demand for agricultural products and water, wastewater re-use will be necessary, which will pose a risk for virus pollution of the environment and subsequent affects viral transmission (Sano et al. 2016). More than 100 species of enteric viruses have been identified in human feces and in sewage such as astroviruses, caliciviruses, enteroviruses, enteric adenoviruses, and rotaviruses (Fernandez-Cassi et al. 2018; Hoque et al. 2019; Metcalf et al. 1995; Gerba et al. 2018). Documenting viral prevalence and diversity in sewage may be a useful method for monitoring viruses circulating in the community (Smith et al. 2016). However, their detection by PCR approaches requires previously identified sequences for primer design and will not detect more distant viruses.

Metagenomics refer to the description of all nucleic acids sequences present in a sample (Forbes et al. 2017). Most of human enteric viruses have small RNA genomes making their detection difficult. Indeed, their relative abundance if compared to bacteria, phage, parasites present in the environment challenge their detection and identification (Cotten and Koopmans 2016; Nieuwenhuijse and Koopmans, 2017; Adriaenssens et al. 2018). Thus, viral metagenomics is generally performed by removing as much host and bacteria as possible followed by nuclease treatment to remove free nucleic acids (Kim et al. 2017; Nieuwenhuijse et al. 2017). Detection of viruses can be performed after de novo assembly of short-read data into longer sequences (contigs) followed by a variety of computational methods for detecting known and novel viral sequences (Cotten et al. 2014; Cotten et al. 2016; Oakeson et al. 2017). However, each sample type is unique and methods need to be adapted to account for origin and to address the metagenomic objective. A concrete example was provided by the analysis of samples collected during the Tara oceans expedition (Alberti et al. 2017). The strategy applied allowed generation of data from a variety of organisms, including viruses and plankton from oceanic samples collected worldwide. Their approach showed the importance of using separate processing steps to analyze the different compartments of a volume of (sea)water. To identify human enteric viruses in complex samples such as stool or sewage samples, a method able to select these small particles resistant to acidic conditions, to eliminate bacteria and to decrease as much as possible background genomes such as phage genomes will be useful.

The aim of the current study was to evaluate methods for human enteric virus detection using metagenomics with a focus on norovirus. Noroviruses have great genomic diversity and they are divided into seven genogroups and many genotypes based upon genomic sequence phylogeny (de Graaf et al. 2017). Three of these genogroups (GI, GII and GIV) infect humans, and constitute the principal agent of acute gastroenteritis worldwide. Importantly for environmental research norovirus are excreted at high concentrations by infected individuals and they are highly persistent (Atmar et al. 2018). Their concentrations in sewage, that may vary among countries, are usually high and their presence in waters constitutes a major public health issue nowadays (Sano et al. 2016; Schaeffer et al. 2018). To specifically enrich our metagenomics libraries in norovirus sequences, we selected three protocols that take advantage of known properties of these viruses. The first method uses SureSelect target enrichment (Agilent) with probes matching human norovirus sequences. The second method comprises pH variations, based on norovirus capsids isoelectric point and on their resistance to both high and low pH. The third method is based on norovirus binding to glycans structures that are present in human but also pig mucins.

## Materials and Methods

### Samples

**Fig. 1:**
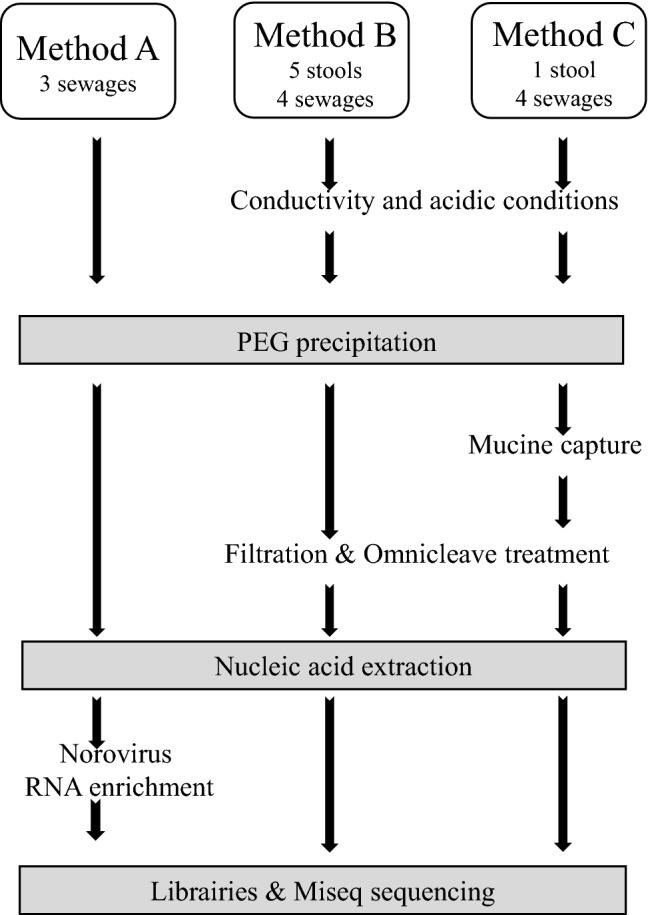
Schematic of sample processing and sequencing used in this study. The three main frames on the top contain the method names (A, B and C), the number and the type of sample treated with each protocol. Key steps common to all protocols are represented in gray rectangle

Five human stool samples (sample 570, 287, 5, 581, 582) positive for norovirus collected between January 2008 and October 2016 were used as a 10% suspension in water. Four samples were analyzed following method B and one sample following method B and C (stool sample 570) (Fig. 1). Seven raw sewage samples were collected from a gathering point upstream of any process between January 2014 and February 2017 from different sewage treatment plants located in the southern part of Brittany (France). Three sewage samples (sample 1777, 1797, 1854) were analyzed using method A and the remaining four samples (sample 1887, 1919, 1920, 1927) were analyzed using method B and C.

### Method A: Polyethylene Glycol Precipitation and Norovirus SureSelect Target

Sewage samples were concentrated using a polyethylene glycol precipitation method (PEG) as previously described (Lewis et al. 1988; Schaeffer et al. 2018). Briefly, 40 mL of sample were mixed with 10 mL of a 50% PEG 6000 solution (Sigma-Aldrich, St Quentin France) (Fig. 1). After gentle agitation overnight at 4 °C, the mixture was centrifuged for 1.5 h at 13,500 × g and the pellet was suspended in 3 mL of 0.05 M glycine buffer (pH 9). After nucleic acid extraction as described below, norovirus RNA sequence were enriched using the SureSelect target enrichment specific array during the library preparation (details in the library preparation paragraph) (Brown et al., 2016).

### Method B: Acidic Treatment and PEG Precipitation

To optimize human enteric viruses recovery, viruses were acid adsorbed to organic or inorganic particles present in the samples by increasing the conductivity of stool suspension or sewage samples to 2000 µS by addition of 5 M sodium chloride at pH 3 (Mullendore et al. 2001), and then concentrated using PEG precipitation as described above (Fig. 1). After centrifugation, the pellet was suspended in 3 mL of 0.05 M glycine buffer (pH 9), and filtered through 5, 1.2, 0.45 and 0.22 µM filters (Minisart NML 17594, NML17593, PES16533, PES16532). Then, the filtrate was incubated with 2000 Units of OmniCleave Endonuclease (Epicentre, Madison, USA) for one hour at 37 °C to eliminate free nucleic acids, followed by nucleic acid extraction.

### Method C: Porcine Gastric Mucin (PGM) Capture

Samples were prepared following method B and after incubation with the OmniCleave Endonuclease, porcine gastric mucin (PGM) capture was applied (Fig. 1). Type III PGM (7.5 mg/mL, Sigma, France) was conjugated to MagnaBind TM carboxyl-derivatized beads (Pierce Biotechnology, Rockford, IL, USA) according to the manufacturer’s protocol. Subsequently, 100 µL of PGM-bead suspension was mixed for 1 hour at room temperature with 3 mL glycine buffer PEG sewage concentrate (Tian et al. 2008). After capturing the beads using a magnetic rack, the supernatant was discarded and the beads were released and suspended in 1 mL of sterile water. This volume was further diluted in the lysis buffer for nucleic acid extraction as described below.

### Nucleic Acid Extraction

Nucleic acids were extracted from sample concentrates prepared using the three methods by adding 10 mL of the chaotropic agent guanidine thiocyanate reagent lysis buffer (bioMerieux, Lyon, France) and incubation for 10 min at room temperature. Then, for samples prepared using Method C, the supernatants were transferred to new tubes after bead capture using the magnetic rack. Then, 140 µL of paramagnetic silica bead suspension was added (NucliSens kit, bioMerieux) to all the tubes and further incubated for 10 min at room temperature. The beads were captured using the magnetic rack and the volume was reduced to 2 mL for further extraction and purification steps as recommended (Schaeffer et al. 2018). A final step of RNA cleaning and concentration step was performed using a Zymo-spin column (RNA Clean & Concentrator, Zymo Research, Irvine, USA). The final step was a DNAse treatment for 30 min at 37 °C with 5 Units of Turbo DNAse, (Ambion, ThermoFisher Scientific, France) (method B and C).

### Norovirus Quantification

A microfluidic-based digital one-step RT-PCR, that allow quantification without external calibration curves was performed using primers and probes targeting the ORF1-2 region (Polo et al. 2016). Positive and negative controls were included in each series, and quantification was calculated using the Poisson distribution (QuantStudio™ 3D Analysis Suite™ Cloud Software, version 3.0.3; ThermoFisher). The final result was expressed as RNA copies/µl.

### Library and Sequencing

For method A, cDNA were synthesized using Superscripts II and random primers according to the manufacturer protocol (Life Technologies). The second-strand cDNA synthesis was performed using 5 U of Klenow (Invitrogen) polymerase in a final volume of 30 µL followed by SureSelect enrichment (Agilent) with the RNA bait design previously designed (Brown et al. 2016). NGS librairies were prepared using a SureSelect^XT^ Illumina paired-end sequencing library protocol (Agilent).

For methods B and C, after cDNA synthesis using SuperScript II and random primers according to the manufacturer's protocol, the second-strand DNA was synthesized according to the manufacturer's protocol (New England BioLabs). NGS libraries were prepared using the NEB Next Ultra DNA Library Prep Kit for Illumina (New England BioLabs) according to the manufacturer’s instructions.

Sequencing was performed on the Illumina MiSeq platform, with Phage *PhiX174* added to samples to standardize the runs.

### Sequence Analysis

Illumina adapters were removed from the raw reads and resulting reads were trimmed using QUASR (Watson et al. 2013) from the 3′ end to reach a median Phred score ≥ 35, which means a base call accuracy between 99.9% and 99.99%. Reads shorter than 80% of the original read length were discarded.

#### General De Novo Assembly

De novo assembly was performed with quality-controlled reads using SPAdes v.3.10.1 (Bankevich et al. 2012). A variety of assembly conditions were examined but in general assembly with no error correction or read normalization yielded the largest initial contig set. Virus specific contigs were identified using Usearch (Edgar 2010), against a set of 39 virus family or subfamily specific protein databases and virus family specific contigs were further assembled into larger contigs using mapping of the contigs against the closest identified full genome. A penultimate consensus genome was generated from the contigs. A final check of the genome was performed by mapping all quality-controlled reads to the penultimate consensus genome and a final majority nucleotide consensus genome was generated. All expected reading frames were examined, any disruption was checked and resolved by consulting the original reads across the query site.

#### Virus Family Specific De Novo Assembly

All virus family-specific reads were then harvested by mapping to a comprehensive set of all sequences > 500 nt available for that family in GenBank using Bowtie2 (Langmead et al. 2012). The resulting virus family-specific reads were de novo assembled using SPAdes v.3.10.1 (Bankevich et al. 2012). Contigs shorter than 500nt were removed from subsequent analysis and contigs with coverage below 10 (determined by Bowtie2 mapping) were carefully examined to avoid assembling contigs with varying coverage. Further assembly and genome checking were performed as described above.

## Results

**Fig. 2:**
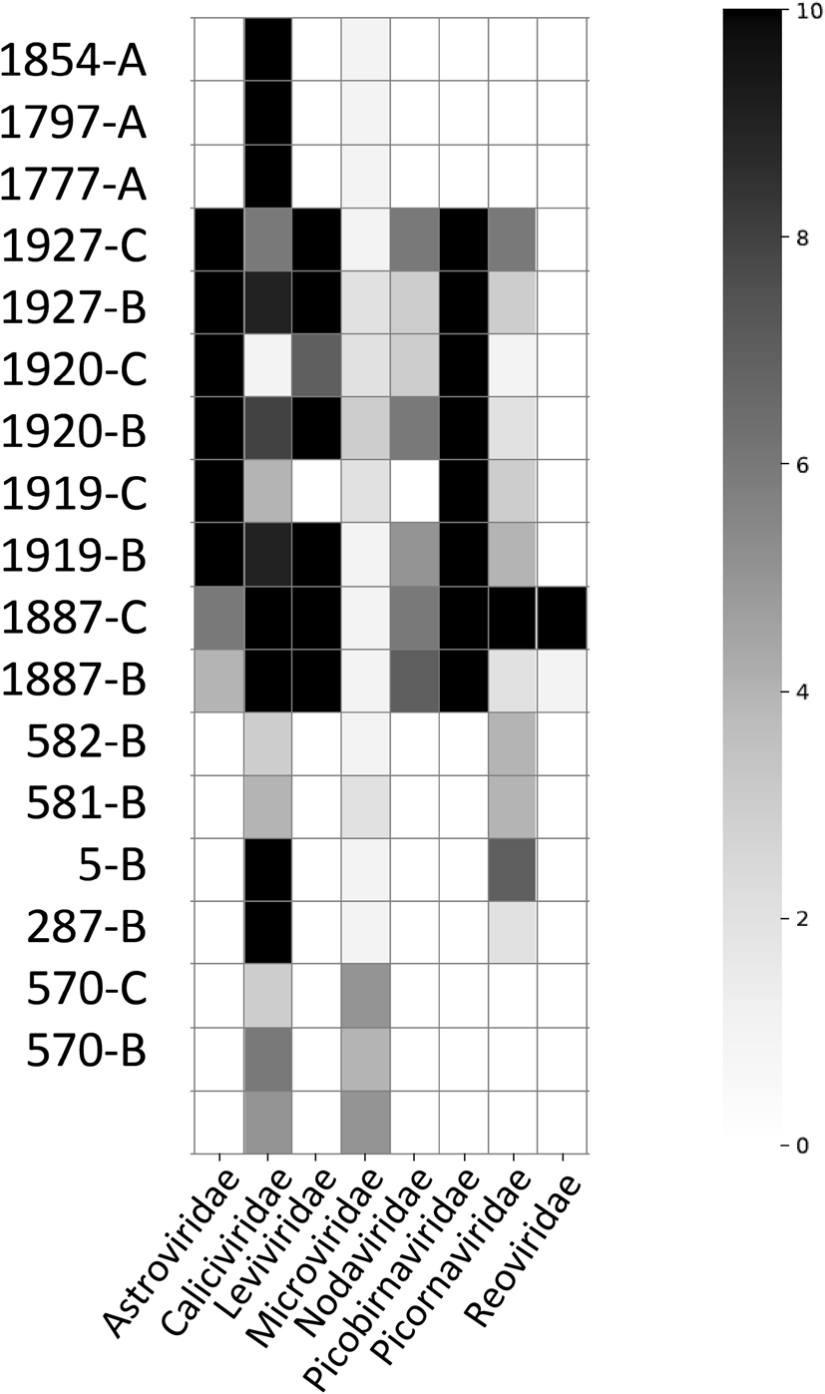
Heat map of larger viral contig yield. Quality-controlled short-read data were de novo assembled into large contigs and contigs were classified at the protein level by comparison with virus family-specific reference sets (see Methods). The numbers of contigs in each sample with > 60% protein identity and 500 nt minimum contig length were plotted in a heat map. The colorbar to the right indicates number of contigs detected per sample in each virus family

One objective of this work was to explore three biochemical and nucleic acid enrichment methods in sample preparation for viral NGS (Fig. 1). Overall, large contigs matched to one of these eight viral families (*Astroviridae, Caliciviridae, Nodaviridae, Leviviridae, Microviridae, Picornaviridae, Picobirnaviridae* and *Reoviridae*) (Fig. 2). These families were detected in multiple samples and we focused on these families for the remaining analyses. No sequence with homology to the Hepatovirus genus or *Hepeviridae* family were detected.

### Norovirus Concentrations in Sewage Samples

Quantification performed on four samples displayed comparable concentration (around 10^3^ RNA copies (c)/uL) for norovirus (Table 1). Norovirus GI was detected in all samples, with concentrations 10 to 100 times lower compare to norovirus GII concentrations. After PGM capture (method C) norovirus concentrations was similar for three samples, but was lower for sample 1920, both for genogroup I and II (Table 1).

**Table 1:** Norovirus concentrations in wastewater samples used for this study and corresponding calicivirus reads

Sample	Method	Norovirus*		Calicivirus
		GI	GII	reads
E1777	A	3	497	NA
E1797	A	64	39	NA
E1854	A	3	37	NA
E1887	B	7	219	2507
	C	2	223	13,548
E1919	B	19	361	3242
	C	6	346	10,635
E1920	B	20	466	10,406
	C	3	*81*	3962
E1927	B	26	140	1750
	C	11	637	2777

*NA* data not collected

*Concentrations obtained using digital RT-PCR are expressed as RNA copies/µL for genogroup I (GI) and II (GII)

**Number of reads mapped on contigs > 500nt using Bowtie2

### Utility of Target Enrichment (Method A) to Study Norovirus Diversity

Targeting norovirus sequences using the norovirus enrichment capture allowed the recovery of long fragments with good coverage as the lowest count was 8.225 (Table 2). Almost complete genomes were obtained for seven GII strains and for two GI strains. Half of a GIV.1 norovirus sequence was also obtained. Another advantage of this approach is the sensitivity as the full-genome sequence for GI strain was obtained from sewage samples 1777 and 1854, having both a quite low concentration of 3 RNAc/µL.

**Table 2:** Norovirus strains identified in sewage samples using method A

Sample	Contig length	Genotype*	Coverage**
E1777	7278	GIIP21-GII.3	19.586
	7484	GIIP7-GII.6	99.576
	6828	GIIPe-GII.4syd-v2	23.144
	7497	GIIPe-GII4syd	21.732
	7648	GIP3-GI.3	16.817
E1797	7343	GIIP17-GII.7	85.503
	7431	GIIP7-GII.6	47.253
	3467	GIP9-GI.9	12.576
	7679	GIPb-GI.6	71.396
E1854	1582	GIIP16-GII.13	12.72
	6509	GIIP17-GII.17	8.225
	1221	GIIPg	8.663
	7641	GIPb-GI.6	15.532
	3687	GIV	12.327

*Sequences were identified using the online norovirus genotyping tool v2.0 (Kroneman et al. 2011)

**Coverage was calculated using Bowtie2 (Langmead et al. 2012)

### Impact of PEG Precipitation (Method B) on Virus Sequence Yield

Viruses were first concentrated using high-molecular weight PEG precipitation for both stool and sewage samples. PEG has been used to concentrate enteric virus in oysters and water samples (Lewis et al. 1988) and when applied here, it was efficient in recovering long norovirus contigs, with five complete norovirus genomes obtained. An additional advantage of the PEG approach when combined with random primed deep sequencing was the detection of other enteric viruses present in the samples such as coxsackievirus in stool samples (Table 3), sapovirus, astrovirus and rotavirus in sewage samples (Table 4).

**Table 3:** Norovirus and other human virus detected in stool samples using method B

Sample	Virus	Contig length	Genotype
S570	Norovirus	7609	GIIPe-GII.4 syd
S287	Norovirus	7542	GIIP21-GII.3
	Norovirus	1104	GIIP17-GII.17
	Enterovirus	653	coxsackievirus A9
S5	Norovirus	7390	GIIP4NewOrleans-GII.4syd
	Norovirus	3399	GII.P21-GII.3
	Enterovirus	1387	coxsackievirus A9
S581	Norovirus	7528	GIIP17-GII.17
	Enterovirus	1421	coxsackievirus A9
S582	Norovirus	7536	GIIP17-GII.17
	Enterovirus	1363	coxsackievirus A9

**Table 4:** Human enteric viruses detected using method B and C

Sample	Method B			Method C		
	Virus	Contig	Genotype	Virus	Contig	Genotype*
S570	Norovirus	7609	GIIPe-GII.4	Norovirus	6976	GIIPe-GII.4
E1887	Sapovirus	7414	GI.2	Sapovirus	7491	GI.2
	Norovirus	3241	GIIP22-GII.17	Norovirus	2366	GII.P22
	Norovirus	2961	GII	Norovirus	2129	GII
	Norovirus	2996	GII.P7-GII.17	Norovirus	3126	GII.P17-GII.17
				Norovirus	3024	GII.P17-GII.6
	Rotavirus	836		Rotavirus	3283	G9[P8]
	Astrovirus	3860	Type 2	Astrovirus	6519	Type 2
				Husavirus	494	Sp.16915_89
E1919	Norovirus	4598	GII.P16-GII.2	Norovirus	1028	GIIP16
	Norovirus	2692	GII	Norovirus	3004	GII.P16-GII.4 syd
	Sapovirus	1209	GII.3			
	Astrovirus	6810	Type 1	Astrovirus	6412	Type 1
E1920	Norovirus	2239	GII	Norovirus	510	GII.2
	Norovirus	332	GI–GI.3			
	Astrovirus	6782	Type 1	Astrovirus	1878	Type 1
E1927	Norovirus	944	GII.2	Norovirus	631	GII.Postdam
				Norovirus	616	GII
	Norovirus	442	GI	Norovirus	631	GI–GI.3
				Sapovirus	571	GI.1 Seoul
	Astrovirus	1878	Type 1	Astrovirus	6588	Type 5
				Astrovirus	3948	Type 1
				Aichi virus	2692	Type 1

*Strain identification was assigned using the online Norovirus genotyping tool v2.0 (Kroneman et al. 2011)

### Impact of Mucin Capture (Method C) on Virus Sequence Yield

**Fig. 3:**
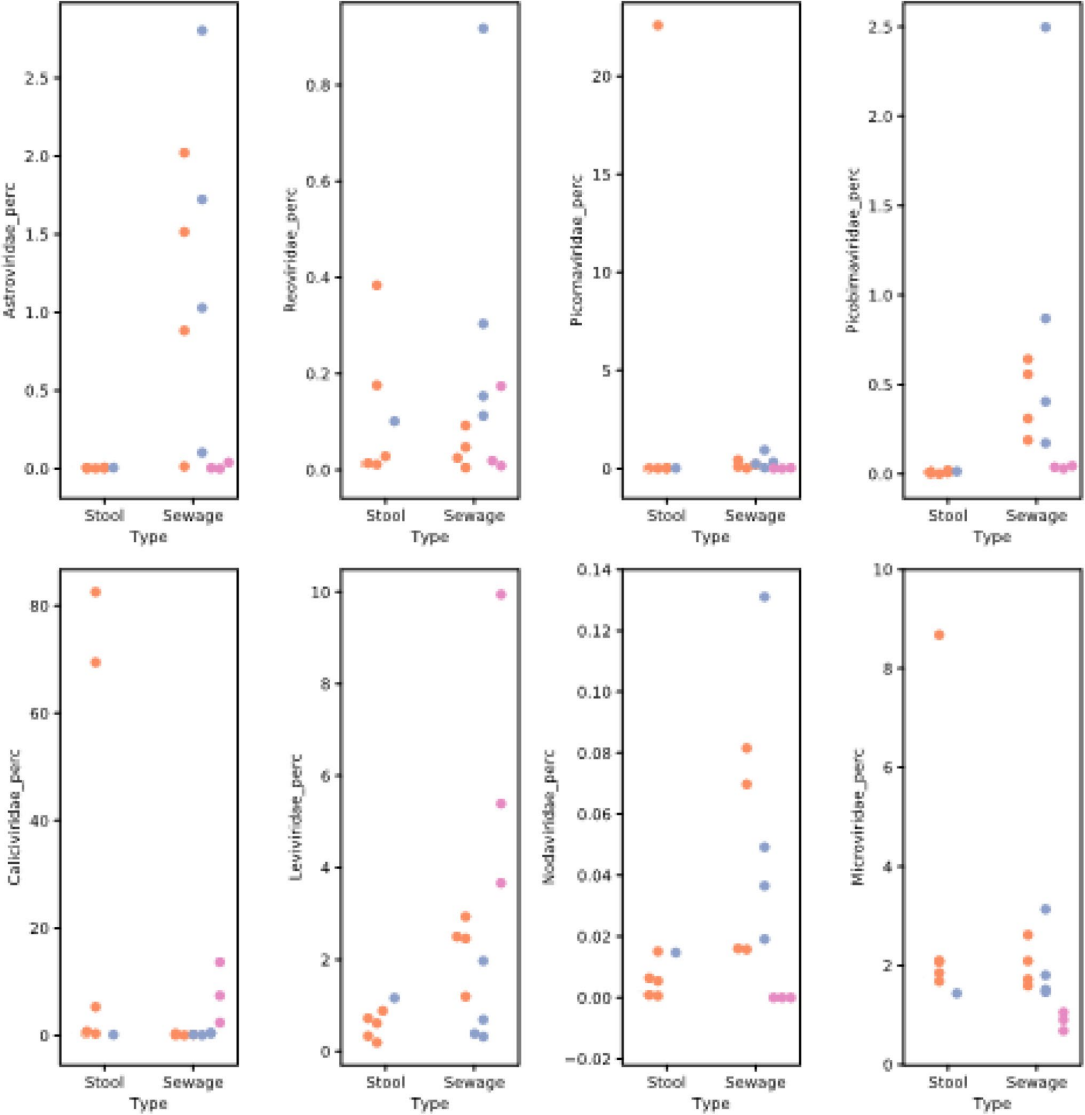
Scatter plot of reads identified for each of 8 virus families (percentage of total reads). Quality-controlled reads for each of the 18 sample/conditions were mapped to virus family reference sets (see Methods) for *Astroviridae, Caliciviridae, Nodaviridae, Leviviridae, Microviridae, Picornaviridae, Picobirnaviridae* and *Reoviridae*. The number of reads identified in each family was plotted as percentage of the total quality-controlled reads for that read set. Data sets were separated into sample types (stool or sewage) and colored according sample treatment (method A: pink, method B: orange and method C: blue)

We aimed to investigate if a mucin capture step could provide enrichment of norovirus and other enteric virus materials. Calicivirus read numbers increased after mucin capture except for sample1920 for which norovirus GI and GII concentrations were also lower suggesting a failure in one purification step or a selection of some norovirus strains by the PGM (Table 1). No reproductible impact of the mucin capture on contig lengths was observed. Examining the lengths of norovirus contigs as a measure of success, four norovirus contigs were longer with mucine capture, while six norovirus contigs showed reduced lengh with mucin capture (compared to contig yield without PGM capture, Method B) (Table 4). An unexpected observation was the impact of this treatment on a number of other viruses as shown by the viral family-specific read yields for eight virus families. For *Astroviridae, Reoviridae, Nodaviridae* and *Picobirnaviridae* families, the yield of specific reads (as a percentage of total reads for that sample) was increased, as reported in upper panels of Fig. 3 comparing orange markers (method B) to blue markers (method C). This was confirmed by the identification of a complete genome for a human rotavirus A genotype G9-[P8], and for astrovirus (full genome for one strain and four additional strains identified) compared to method B (Table 4). In contrast, the yields of *Leviviridae* and *Picornaviridae* families remained similar or slightly reduced. A large amount of the phage *PhiX174 (*a member of the *Microviridae* family*)* was added as a carrier after the PGM capture, thus we cannot make conclusion about the impact of PGM on the *Microviridae* family.

## Discussion

This work aimed to generate enteric virus sequence data from human stool and sewage samples, with a special interest in norovirus. There are several challenges when applying metagenomics deep sequencing to describe the total virome of sewage samples (Bibby et al. 2013, Adriaenssens et al. 2018). One challenge is the high levels of dilution of human enteric viruses and the presence of a large variety of other microorganisms, plant, algae, chemical and organic compounds. Indeed, in non-outbreak settings, human enteric viruses are present at lower concentrations compared to bacteria or phages. In a previous study, we demonstrated that at least 1-4% of the population need to be infected to detect hepatitis E virus in raw sewage from a small sewage treatment plant, showing the potential to rapidly detect outbreak in the population (Miura et al. 2016). Another challenge of sewage virome characterization is to obtain a representative sample of the viral load and diversity from the wastewater. Concentration of large volumes of water are currently used to decrease the sampling variability and increase the sensitivity threshold of the assays (Lewis et al. 1988; Schaeffer et al. 2018; Fernandez-Cassi et al. 2018). However, this may lead to increase concentration of inhibitors of the enzymes used for molecular assays, as human sewage may contain detergents, medicine, food additives, food waste and other chemicals and thus some purification steps are needed (Hata et al. 2017). Composite samples obtained over 24 h, as used for this study, may be a convenient way to overcome some of these problems as it can be representative of what is entering in the sewage treatment plant without the need of large volumes. Different methods previously developed for sewage, water or shellfish analysis use the PEG precipitation that help to concentrate biomolecules by altering their hydration from solution and viruses (Lee et al. 1981; Lewis et al. 1988; Metcalf et al. 1995; Jiang et al.^1992^). This easy-to-use method was proposed for the first assay able to detect norovirus in stool (Jiang et al. 1992) and in preliminary test we verified that it helped to recover the complete norovirus genome from stool samples (data not shown). As human enteric viruses tend to aggregate or to bind to different types of particles, including bacteria, we applied an elution step based on pH variations under controlled conductivity conditions (Miura et al. 2013; Samandoulgou et al. 2015; Mullendore et al. 2001; da Silva et al. 2011). This added step may explain the higher efficacy to obtain long sequence of human enteric viruses compared to a published study that failed to identified norovirus strains (Hjelmso et al. 2017).

Capsid structures of a number of human enteric viruses including norovirus and rotavirus have been found to interact with the mucin family of glycoproteins due to the presence of binding sites for the complex carbohydrates on mucin (Le Pendu et al. 2014). This binding affinity was proposed to select norovirus particles from water samples using human histo-blood group antigen or PGM that present similar structure as it may increase the specificity by capturing non-damaged capsid and the sensitivity of detection by facilitating inhibitor removal (Zhou et al. 2017; Tian et al. 2008). In this study, PGM capture increased norovirus concentrations in the nucleic acid extracts, however, no difference was observed in terms of contig lengths or diversity of identified sequences. Combining PGM and HBGA type-B antigen could have help to improve this approach (Tian et al., 2017). One beneficial impact of the PGM capture was the detection of full genome of a rotavirus genogroup A genotype G9-[P8], confirming the binding affinity of rotavirus strains to glycans (Hu et al. 2018). When compared to method B (PEG without mucin capture), rotavirus was identified but full genome was not obtained, suggesting that PGM combined with random primed deep sequencing would be superior to detect full genomes of norovirus and rotavirus. It was more surprising to find a beneficial impact of PGM capture on astrovirus, with an increased diversity of strains identified. The PGM capture led also to the characterization of a few sequence of husavirus strain. Some husaviruses have been described as a novel virus family within the order Picornavirales, and are common in human fecal material (Oude Munnink et al. 2015). However, very little is known about astrovirus or husavirus PGM interactions, which may be due to non-specific interactions such as sialic acid recognition or other glycan affinity to viral capsid structure (Shanker et al. 2017; Hu et al. 2018). Adding purification steps enhance the risk to lose viruses as observed for one sample and also to introduce some bias of selection. Furthermore, when dealing with complex samples with a mixture of low amounts of viral contaminantions such as sewage, this may be an issue which need to be further investigated. Three biological replicates were found to be an useful approach to minimize potential biases and to give more confidence in analyzing aquatic viromes (Kim et al. 2017).

One objective of this work was to evaluate norovirus diversity present in stool and sewage samples. Some works based on a metabarcoding approach targeting the ORF2 portion of norovirus genome were successfully applied to sewage samples, but this approach limits the identification of strains that are not amplified by published primers (Oshiki et al. 2018; Fumian et al. 2019). In our work, we aimed to identify long fragments to be able to capture the diversity of strains circulating in the local population. The SureSelect target enrichment (method A) gave the highest number of full genome for several norovirus strains despite similar norovirus concentrations when compared with sewage samples used (Brown et al. 2016). This method was the only one able to yield norovirus GI strain identifications, important criteria for environmental sample analysis considering the importance of these strains transmission through the environment (Le Guyader et al. 2012; Verhoef et al. 2015). Although the SureSelect target enrichment array is designed to capture *Caliciviridae* sequences, these targeted sequences were still less than 1% of the total reads after capture, suggesting non-specific binding. Since the binding and wash conditions are propriety to the manufacturer and the actual bait concentrations are not provided, specificity improvements are limited. This method also allowed to identify a norovirus GIV.1 strain. This genogroup is sporadically detected in sewage samples, and may be under-appreciated as a cause of gastroenteritis, presumably because only a small number of sequences are available in GenBank causing difficulties in primer design (Sima et al. 2011; La Rosa et al. 2012). The two other methods also allowed to characterize some norovirus sequence, all of them being already reported in the NoroNet network (van Beek et al. 2018). Beside the ubiquitous GII.4 strains that have been reported worldwide, it was interesting to detect the GII.P17-GII.17 strain that caused several gastroenteritis outbreaks in multiple countries during this sampling period (Matsushima et al. 2015; Koo et al. 2017). In this regard, a complete characterization of detected viruses is important to identify new strains and thus to help risk manager to take measure to prevent further distribution (Cocolin et al. 2018).

A limitation of this study lies in the small sample size tested. Ideally, it would be more informative to have a larger number of samples being tested for the same approach to evaluate the method efficiency across sample variability. Sequence contents of raw sewage reflect the composition of the microbiome of local population and the diversity of all pathogenic or non-pathogenic bacteria or viruses circulating in the community at a larger extent than individual samples (Newton et al. 2015; Sano et al. 2016). To access this sequence information, we need to develop reproducible, simple, fast and easy to apply methods. The viral agnostic metagenomics approach is still an expensive approach when considering the library preparation or sequencing runs, but all the steps of the methods described here can easily be performed in environmental laboratory already performing PCR analysis as it just needs a centrifuge and basic equipment.

## Data Availability

The short-reads data for this study has been deposited in the European Nucleotide Archive (ENA) http://www.ebi.ac.uk/ena/data/view/PRJEB31600 and to Genbank with the following (temporary) accession number: from MK789654 to MK789656 for NoV GI, from MK907785 to MK907802 for NoV GII, MK726262 for NoV GIV.
